# Enhanced Photoelectrochemical Performance of BiVO_4_ Photoanodes Through Few-Layer MoS_2_ Composite Formation for Efficient Water Oxidation

**DOI:** 10.3390/ma18245639

**Published:** 2025-12-15

**Authors:** Deepak Rajaram Patil, Santosh S. Patil, Rajneesh Kumar Mishra, Sagar M. Mane, Seung Yoon Ryu

**Affiliations:** 1 Department of Physics, Dongguk University, Seoul 04620, Republic of Korea; 2 Photoenegy Harvesting and Conversion Technology (PHCT), Dongguk University, Seoul 04620, Republic of Korea; 3 Department of Research and Development, Dr. Vishwanath Karad MIT World Peace University (MIT-WPU), Kothrud, Pune 411038, India; 4 Department of Physics, Yeungnam University, Gyeongsan 38541, Republic of Korea; 5 Department of Fiber System Engineering, Yeungnam University, Gyeongsan 38541, Republic of Korea

**Keywords:** hydrothermal method, BiVO_4_-MoS_2_, hybrid photoanodes, PEC-WS

## Abstract

Photoelectrochemical water splitting (PEC-WS) provides a sustainable route to transform solar energy into hydrogen; however, its overall efficiency is constrained by the inherently slow kinetics of the oxygen evolution reaction. Bismuth vanadate (BiVO_4_) is considered an attractive visible-light-responsive photoanode due to its suitable band gap (~2.4 eV) and chemical stability; however, its efficiency is restricted by limited charge transport and significant charge carrier recombination. To overcome these limitations, BiVO_4_–MoS_2_ (BVO–MS) heterostructures were synthesized through a simple in situ hydrothermal approach, ensuring robust interfacial coupling and uniform dispersion of MS nanosheets over BVO dendritic surfaces. This intimate contact promotes rapid charge transfer and improved light-harvesting capability. Structural and spectroscopic analyses confirmed the formation of monoclinic BVO with uniformly integrated amorphous MS. The optimized BVO–MS10 electrode delivered a photocurrent density of 4.72 mA cm^−2^ at 0.6 V vs. SCE, approximately 5.3 times higher than pristine BVO, and achieved an applied bias photon-to-current efficiency of 0.49%. Mott–Schottky analysis revealed a distinct negative shift in the flat-band potential for BVO–MS10, indicative of an upward movement of its conduction band and the establishment of a strong internal electric field that enhances charge separation and interfacial electron transport. These synergistic effects collectively endow the in situ engineered BVO–MS heterostructure with superior PEC water oxidation performance and highlight its promise for efficient solar-driven hydrogen generation.

## 1. Introduction

Photoelectrochemical water splitting (PEC-WS) represents an exceptionally effective strategy for directly converting plentiful solar light into clean and storable chemical fuels, offering a sustainable pathway without carbon emissions [1,2,3]. Among the two half-reactions involved, the anodic water oxidation (oxygen evolution reaction, OER) is generally acknowledged as the rate-determining step because it involves a complex four-electron transfer process to generate molecular oxygen [4,5]. In recent decades, a wide range of materials have been investigated as photoanodes to overcome this kinetic bottleneck. Metal oxides, including TiO_2_ [6], WO_3_ [7], Fe_2_O_3_ [8], Ta_3_N_5_ [9], ZnO [10], etc., have emerged as the most widely investigated class of photoanodes for PEC-WS owing to their excellent chemical stability in aqueous electrolytes, cost-effectiveness, and environmental compatibility. Nevertheless, relatively wide band gaps of most of the metal oxides restrict efficient utilization of the solar spectrum, thereby limiting overall conversion efficiency. To address this issue, it is essential to develop new photoanode materials with optimal band gaps that can effectively harness visible light while maintaining high stability and activity. Among various potential materials, BiVO_4_ (BVO) with a monoclinic scheelite structure has garnered considerable interest as a potential visible-light-responsive photoanode [4,11,12]. With a moderate band gap of approximately 2.4 eV, good chemical robustness, and an optimal valence band alignment that supports water, BVO offers an attractive balance of properties. Nevertheless, low carrier mobility and the short diffusion length of holes, which are mainly caused by intrinsic defects and recombination losses, greatly hinder efficient charge separation and transport, leading to PEC performance that remains far below its theoretical potential.

To address the inherent drawbacks of BVO, including fast charge recombination and restricted charge diffusion, researchers have increasingly adopted the strategy of constructing heterostructures by coupling two or more semiconductors or metals [13,14,15,16,17]. This approach enables more efficient utilization of sunlight, improved electron–hole separation, enhanced carrier mobility, and higher overall photoconversion efficiency. Integrating BVO with photoactive components that possess complementary energy-band structures is particularly effective in promoting its photoelectrochemical performance. Among the various candidates, 2D materials have received significant attention because layered architecture provides continuous charge transport channels that facilitate hole extraction and minimize surface recombination. MoS_2_ (MS) has garnered significant interest as a potential 2D material due to its high specific surface area, adjustable electronic properties, and excellent catalytic activity. The positions of its band edges are found to be well aligned with those of BVO, making MS an excellent co-catalyst for accelerating surface redox kinetics in photoelectrochemical water splitting [18,19,20].

Several studies have shown that combining BVO with MS can improve PEC activity [18,19,20]. However, most previous approaches use post-synthesis coating or physical mixing of MS with already prepared BVO powders or films. These ex situ methods often lead to poor interfacial contact, uneven MS coverage, and particle aggregation, which reduce charge transport efficiency and light absorption. In this work, BVO was in situ grown on the conductive substrate using a single-step hydrothermal process in the presence of dispersed MS. This in situ growth allows both materials to form together, creating a strong and well-connected interface. The close bonding between BVO and MS enhances charge transfer, reduces recombination losses, and ensures uniform MoS_2_ distribution within the BVO structure [18,19,20]. As a result, the obtained heterostructure is more stable, compact, and strongly attached to the substrate compared to those made by physical mixing. This also provides better electron pathways and stronger light-harvesting ability. Using this simple hydrothermal method, we successfully fabricated BVO/MoS_2_ hybrid photoanodes, where MS nanosheets were uniformly decorated on BVO dendrites to boost oxidation reactions. Under AM 1.5 G illumination, the hybrid electrode showed about 5.3 times higher PEC performance than pristine BVO, primarily attributed to enhanced charge carrier density, conductivity, and separation efficiency.

## 2. Experimental

The BiVO_4_-MoS_2_ (BVO-MS) Photoanodes were grown in situ on the fluorine-doped tin oxide (FTO) deposited glass substrate via a facile hydrothermal synthesis without the use of any surfactant. A similar process was repeated, which was utilized in our previous work [13]. Initially, a mixed solvent system was prepared by combining 5 mL of HNO_3_ (70%), 55 mL of DI water, and 5 mL of ethanol (C_2_H_5_OH, 94.5%; DAEJUNG, Siheung-si, Republic of Korea). The solution was magnetically stirred for 5 min to obtain homogeneity. Subsequently, 1.12 mmol of NH_4_VO_3_ and Bi(NO_3_)_3_·5H_2_O were mixed, and stirring continued until the precursors were completely dissolved. Few-layer MoS_2_ (MS) powder was then dispersed in the required ratio (mass concentration @ 5%, 10%, and 15%) to the solution and magnetically stirred for 15 min to ensure uniform distribution. The bulk MoS_2_ purchased from Sigma-Aldrich (St. Louis, MO, USA) was used to develop the few-layer. Here, the liquid phase exfoliation (LPE) method was used to fabricate the few-layered MoS_2_, as explained elsewhere previously [21]. Details about LPE were further provided in the Supplementary Materials. FTO glass substrates were subjected to ultrasonic cleaning successively in acetone, ethanol, and deionized water. After cleaning, the FTO substrates were transferred into a hydrothermal autoclave. The prepared precursor solution was poured into the reactor, which was sealed and heated at 150 °C for 12 h. Following completion of the hydrothermal process, the autoclave was left to cool to ambient temperature, yielding uniform BVO-MS thin films directly grown on the FTO substrate. These films were used as photoanodes for PEC-WS experiments. Following a similar process, the pure BiVO_4_ film was developed and labeled as BVO, while with 5%, 10%, and 15% mass concentrations of MoS_2_, it is labeled BVO-MS5, BVO-MS10, and BVO-MS15, respectively.

The phase composition of the powders was examined using XRD (Malvern Panalytical, Worcestershire, UK). X-ray diffraction measurements were done at room temperature with a Cu Kα source (λ = 1.54056 Å). The 2θ range was kept between 10 to 80° with a step size of 0.052°. The surface morphology and microstructural features were observed by FE-SEM (FE-SEM, model S-4800; Hitachi, Tokyo, Japan). HR-TEM (TEM; JEOL/JEM-F200, Akishima, Tokyo, Japan) was employed to investigate the detailed lattice structure. The surface composition and valence states of the elements were characterized using XPS (Thermo Fisher Scientific (K-Alpha), Seoul, Republic of Korea). This spectrometer was equipped with a monochromatic Al Kα X-ray source (hν = 1486.6 eV). Data processing, background subtraction, and peak fitting were performed using Origin software (v-9.3) with a Shirley background and mixed Gaussian–Lorentzian peak shapes. Optical properties were analyzed using UV-Vis spectroscopy (Agilent Technologies, Cary 5000, Santa Clara, CA, USA), while the photoluminescence (PL) spectra were obtained with a PC spectrofluorometer with an excitation wavelength of 325 nm. PEC-WS activity was assessed in a custom-designed Teflon cell filled with 0.5 M Na_2_SO_4_ electrolyte (pH ≈ 7) utilizing a three-electrode system interfaced with a PARSTAT 3000 potentiostat (AMETEK Scientific Instruments, Oak Ridge, TN, USA). In this setup, the BVO–MS composite acted as the photoanode, and a platinum wire was used as the counter electrode. BVO–MS photoanode (effective area ≈ 0.52 cm^2^) was sealed against the cell with an O-ring to maintain proper electrolyte contact. A front-mounted quartz window enabled illumination from a 300 W xenon lamp (≈100 mW cm^−2^).

## 3. Results and Discussion

**Figure 1:**
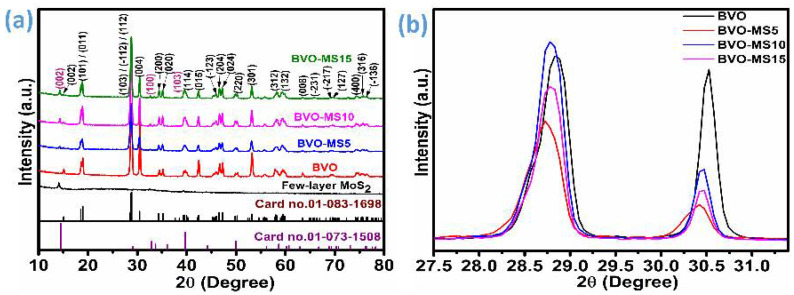
X-ray diffraction of few-layer MoS_2_, BVO, and BVO-MS composites (**a**) X-ray diffraction patterns, (**b**) enlarged view of patterns between 2θ = 27.5 to 31.5°.

The phase structure and crystallinity of the BVO–MS composites were systematically examined using room-temperature XRD, as illustrated in Figure 1a. Diffraction data were collected for all prepared samples over the 2θ range of 10–80° using finely ground powders of the respective composites. The obtained diffraction profiles exhibit distinct and well-defined reflections corresponding to the monoclinic BVO, indicating the preservation of its crystal framework within the composite matrix. No additional diffraction peaks attributable to secondary or impurity phases were detected, suggesting high phase purity. However, noticeable peak broadening was observed across all diffraction patterns, which can be ascribed to the nanocrystalline nature and the reduced coherent scattering domain of the MS component within the composite. The major crystal planes (002), (100), and (103) at 2θ values of 14.10, 32.55, 39.40° corresponding to the MoS_2_ lattice planes were identified, implying that MoS_2_ exists in a few-layered structure within the BVO matrix with hexagonal crystallinity [18]. The intensity of these crystal planes increases as the percentage of the MS component increases. Furthermore, a systematic shift in the major BVO diffraction peaks toward lower 2θ values was observed with the initial incorporation of MoS_2_, indicating lattice expansion or strain effects induced by interfacial coupling or substitute interactions between the two phases. At higher MoS_2_ concentrations, the diffraction peaks initiate shifting back toward higher 2θ angles, suggesting partial lattice relaxation or structural reorganization within the composite framework [18,19]. To understand this shifting with the MoS_2_ fraction, the selective region was plotted in Figure 1b, which shows the crystal planes between a 2θ range of 27.5 to 31.5°. Overall, all the indexed diffraction peaks are in excellent agreement with those of the monoclinic BVO (ICDD card no. 01-083-1698), confirming that the primary crystalline structure of BVO is retained in the BVO-MS composites despite the integration of MoS_2_. Furthermore, the few-layered MoS_2_ peaks are assigned according to the hexagonal phase of MoS_2_ (ICDD card no. 01-073-1508) as illustrated in Figure 1a. The diffraction patterns of the bulk and few-layered MoS_2_ samples after exfoliation are represented in Figure S1. The lattice parameters were tabulated according to the monoclinic and hexagonal crystal systems. Furthermore, the average crystallite size (D) and the microstrain were calculated using the Williamson-Hall method [22].

(1) $$\beta cos\theta =\frac{K\lambda }{D}+\left(strain\right)4sin\theta$$

where β is the line broadening in radians, θ is the Bragg angle, λ represents the wavelength of the X-ray, K is a constant (0.94), and D is the crystallite size of each plane. Microstrain was derived from the plot of βcosθ vs. 4sinθ, which was represented in Figure S2 for all samples. Lattice parameters, average crystallite size, and strain values were summarized in Table S1.

**Figure 2:**
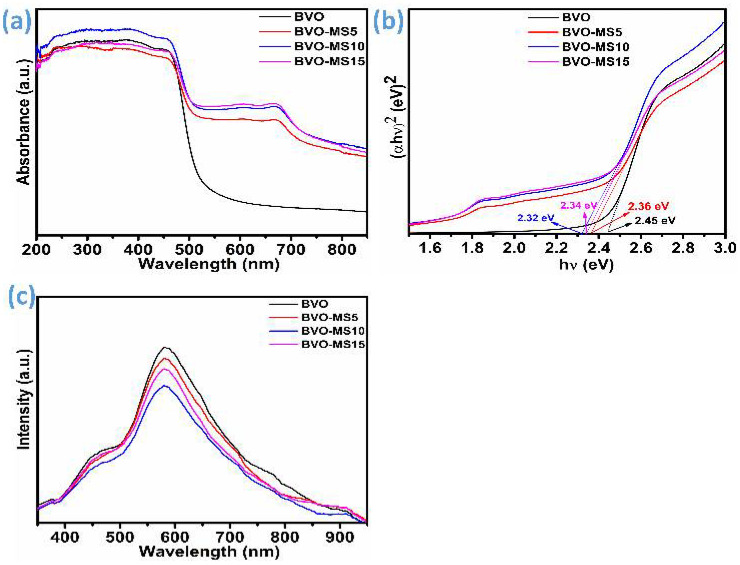
Optical properties of BVO and BVO-MS composites, (**a**) UV-Vis spectra, (**b**) Tauc plot (band gap energy), and (**c**) PL spectra.

The optical behavior of the BVO-MS heterostructures was systematically investigated through UV-vis diffuse reflectance spectroscopy (DRS), as depicted in Figure 2a. The obtained spectra clearly demonstrate that all BVO-MS composites exhibit strong absorption within the visible-light range, particularly between 500 and 600 nm. An additional absorption feature is observed near 670 nm in the optical spectrum of the BVO-MS composite, exhibiting only minor shifts with varying MoS_2_ loading. This absorption band is characteristic of few-layer MoS_2_ structures and originates from electronic transitions at the K-point in the Brillouin zone [19,23]. Such transitions are typically associated with the direct band-to-band excitation within the MoS_2_ lattice, reflecting the preservation of its quasi-two-dimensional semiconducting nature [24]. The subtle dependence of this absorption peak on MoS_2_ concentration suggests that the structural integrity and electronic coupling of MoS_2_ layers within the composite are largely retained, with only limited perturbation from interfacial interactions with the BiVO_4_ matrix. The absorption edge exhibited a distinct red shift, suggesting improved light-harvesting capability with the progressive addition of MS, relative to the pristine BVO sample. This spectral shift indicates a gradual reduction in the optical band gap, which can be attributed to enhanced electronic interactions at the BVO/MS interface. Intimate interfacial contact facilitates efficient charge transfer between the two components, thereby extending the photo response toward longer wavelengths and improving overall light-harvesting efficiency [18,25].

The optical band gap energies were quantitatively determined using Tauc plot analysis, as presented in Figure 2b. The Tauc relation can be given as

(2) $${\left(\alpha h\nu \right)}^{n}=A(h\nu -E_{g})$$

where *α* stands for absorption coefficient, *hν* is the energy of a photon, *E_g_* is the band gap energy, and *A* is a constant. The value of n can be ½ or 2. In this case, the value of *n* is 2, which is considered as per the best linear fitting and previous reference [18]. The pristine BVO sample exhibited a direct band gap of approximately 2.45 eV. Upon incorporation of MS, the band gap values exhibited a decreasing trend for composites containing 5 wt% (2.36 eV) and 10 wt% MS (2.32 eV), suggesting a progressive modification of the electronic band structure due to hybridization between BVO conduction and valence band states with those of MS. Interestingly, a marginal widening of the band gap was recorded for the 15 wt% MS (2.34 eV) composite, which could result from excessive MS loading leading to partial shielding of the active BVO surface or the formation of non-ideal interfaces that hinder charge delocalization. This behavior is unexpected; however, it may arise from reduced interfacial interaction between BiVO_4_ and few-layered MoS_2_ at the higher loading of 15% MS, which could lead to a slight increase in the band gap (2.34 eV). Since the band gap of MoS_2_ or any 2D layered material is known to vary with the number of layers [26], changes in stacking or dispersion at higher content can influence the overall optical response of the composite. A similar kind of behaviour was noted for the composite of ZrO_2_ and S-doped g-C_3_N_4_ [27] in which the band gap varies according to g-C_3_N_4_ concentration. Further, the bandgap can also be tuned by exfoliation and doping of different atoms in g-C_3_N_4_ [28]. In contrast, the 10% MS sample appears to provide the most effective electronic coupling and interfacial charge-transfer interaction with BiVO_4_, resulting in a slightly smaller band gap. It is likely that at this moderate MoS_2_ content, the nanosheets are well dispersed and establish intimate contact with the BiVO_4_ surface. This enhanced interfacial contact can promote stronger electronic interaction and more pronounced band bending, as reflected in the DRS spectra (Figure 2a). These observations collectively confirm that controlled incorporation of MS into the BVO matrix effectively tailors its electronic and optical characteristics, optimizing the heterostructure for superior visible-light-induced photocatalytic efficiency.

Photoluminescence (PL) measurements were employed to further clarify the behavior of light-induced charge carriers and to determine whether they predominantly recombine radiatively or remain effectively separated. The PL responses of the BiVO_4_-MoS_2_ (BVO-MS) series containing different MoS_2_ loadings are presented in Figure 2c. Overall, the spectral shapes of pristine BiVO_4_ and the BVO-MS composites remain comparable; however, noticeable variations arise in the emission intensity as the MoS_2_ concentration changes. The emission feature appears as a broad band centered between approximately 581 and 583 nm, with slight shifts dependent on the MoS_2_ content. Comparable emission characteristics have been reported for BiVO_4_-based heterostructures in earlier studies [29]. Upon introducing MoS_2_ into BiVO_4_, a progressive decrease in PL intensity is observed, indicating a reduction in the radiative recombination rate of photogenerated electrons and holes. From these results, it can be inferred that pristine BiVO_4_ exhibits the most rapid recombination, whereas the BVO–MS10 composition shows the most suppressed recombination among the series. This trend aligns with the general understanding that a weaker PL signal corresponds to more efficient charge separation and reduced electron-hole annihilation [30,31]. The enhancement in charge-transfer efficiency can therefore be attributed to the presence of MoS_2_, which acts as a mediator facilitating spatial separation of carriers. A minor rise in PL intensity in the BVO-MS15 sample suggests a partial decline in interfacial charge separation efficiency at higher MoS_2_ content, likely due to diminished interfacial contact effect that is also consistent with the UV–Vis DRS observations.

**Figure 3:**
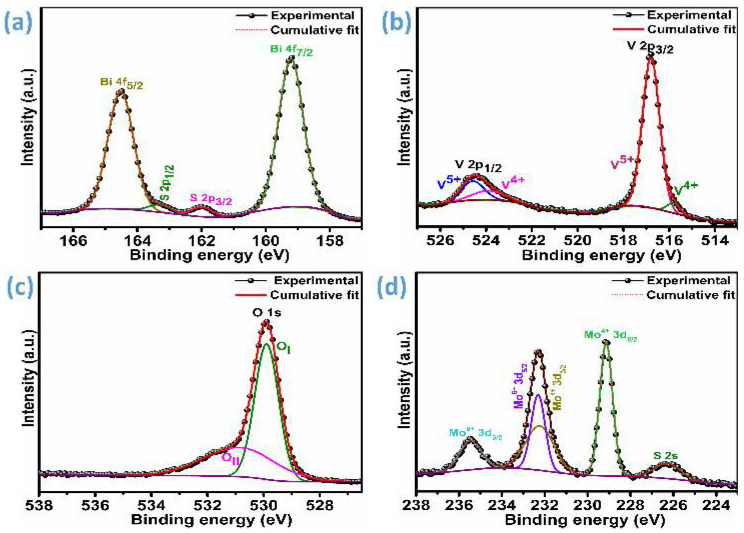
High-resolution XPS spectra of the BVO-MS10 sample, (**a**) Bi 4f & S 2p, (**b**) V 2p, (**c**) O 1s, and (**d**) Mo 3d.

To investigate the surface composition and the electronic configurations of the elements constituting the BVO-10MS heterostructure, XPS measurements were carried out, as illustrated in Figure 3a and Figure S3 (Supplementary Materials). The survey spectrum (Figure S1) distinctly evidences the presence of bismuth (Bi), vanadium (V), oxygen (O), molybdenum (Mo), and sulfur (S), verifying the coexistence of both BVO and MS phases within the heterostructure. The high-resolution Bi 4f spectrum (Figure 3a) exhibits two prominent spin–orbit doublet peaks centered around 159.2 eV and 164.5 eV, corresponding to the Bi 4f_7/2_ and Bi 4f_5/2_ states, respectively. The energy separation of 5.3 eV and binding energy values are consistent with Bi^3+^ species, indicating that bismuth exists in the +3 oxidation state within the BiVO_4_ lattice [19,32,33,34,35]. Additionally, minor features attributed to S 2p are observed at 161.9 eV and 163.2 eV, which correspond to S 2p_3/2_ and S 2p_1/2_, respectively, confirming the successful incorporation of the MS component containing sulfide species [36,37].

The V 2p XPS spectra (Figure 3b) display a distinctly separated doublet, with the main peaks centered around 516.0 eV for V 2p_3/2_ and 524.0 eV for V 2p_1/2_. These binding energies are characteristic signatures of vanadium in the pentavalent oxidation state (V^5+^), confirming that the majority of vanadium species retain their V^5+^ configuration within the BiVO_4_ (BVO) lattice following heterojunction formation [32,33]. Upon spectral deconvolution, additional minor contributions corresponding to V^4+^ species are observed at approximately 515.8 eV (V 2p_3/2_) and 523.9 eV (V 2p_1/2_), indicating partial reduction of vanadium [38]. Quantitative analysis reveals that the predominant portion of the surface vanadium exists as V^5+^, accounting for about 75.7% from the 2p_3/2_ component and 12.3% from the 2p_1/2_ component, with slightly shifted binding energies at 516.8 eV and 524.6 eV, respectively. The persistence of the V^5+^ oxidation state plays a critical role in preserving the intrinsic photoelectronic characteristics of BiVO_4_, ensuring efficient charge separation and sustaining its inherent photocatalytic activity within the heterostructure system [35,36].

The O 1s spectrum (Figure 3c) presents two distinguishable components located near 529.9 eV and 531.0 eV. The lower binding energy peak (O_I_) is attributed to lattice oxygen bound to metal cations (M-O bonds), while the higher binding energy peak (O_II_) corresponds to oxygen species associated with oxygen vacancies or surface-adsorbed hydroxyl groups [13,19]. The O_II_ component indicates an increased density of surface defects, which may act as active sites for charge trapping and facilitate charge transfer across the BVO-MS interface. In the Mo 3d spectrum (Figure 3d), two main peaks appear at 229.1 eV (Mo 3d_5/2_) and 232.2 eV (Mo 3d_3/2_), implying the existence of the Mo^4+^ oxidation state [18]. A small number of Mo^6+^ 3d_5/2_ and 3d_3/2_ states are noted at 232.3 eV and 235.4 eV, indicating the commercial grade MoS_2_ [24]. The presence of the Mo^6+^ species suggests that the MoO_3_ layer has developed. This oxidation can arise either from exposure of the sample to ambient conditions or from partial sulfur depletion during the hydrothermal synthesis, both of which can promote surface oxidation [39]. Furthermore, a subtle signal observed at 226.3 eV, corresponding to the S 2s level, confirms the presence of S^2−^ ions, reinforcing the successful integration of the sulfide phase [19,35]. Collectively, these XPS results unequivocally demonstrate the formation of a well-integrated BVO-MS heterostructure, where the constituent elements retain their expected oxidation states while establishing interfacial electronic interactions. The coexistence of oxygen vacancies and mixed-valence states further indicates the generation of defect-mediated charge transport pathways, which can play a pivotal role in enhancing the heterostructure’s photoelectronic and catalytic performance.

**Figure 4:**
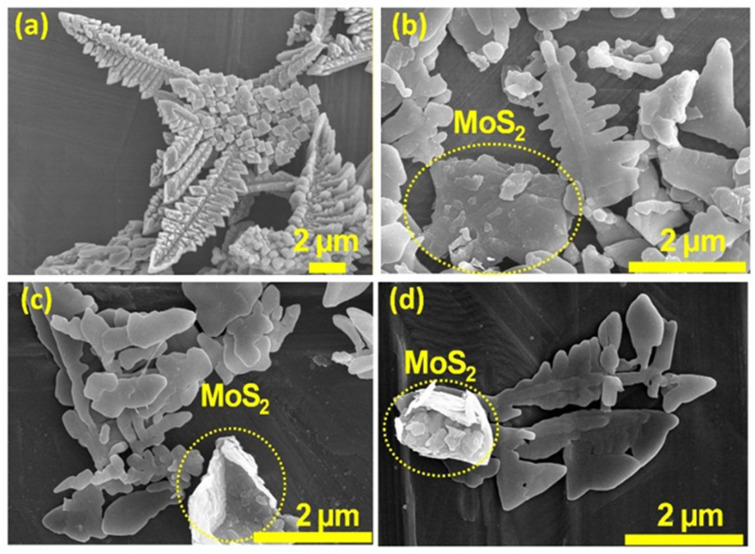
FE-SEM images of BVO and BVO-MS composites at higher magnification, (**a**) BVO, (**b**) BVO-MS5, (**c**) BVO-MS10, and (**d**) BVO-MS15.

The surface morphology and structural characteristics of the BVO and BVO-MS heterostructures were systematically examined through field-emission scanning electron microscopy (FESEM), as illustrated in Figure 4 and Figure S4. The pristine BiVO_4_ (Figure 4a and Figure S4a) displays a distinctly dendritic microstructure composed of interconnected rod-like branches organized into a three-dimensional (3D) hierarchical framework. This intricate dendritic network inherently provides an extensive specific surface area and continuous electron/ion diffusion channels, both of which are highly favorable for accelerating interfacial electrochemical reactions and facilitating efficient charge transport. Following the introduction of the MS component (Figure 4b and Figure S4b for BVO-MS5, Figure 4c and Figure S4c for BVO-MS10, and Figure 4d and Figure S4d for BVO-MS15), the fundamental dendritic configuration of BVO remained largely preserved, indicating that the formation of the heterostructure did not disrupt the intrinsic crystal growth habit of BVO.

However, a noticeable thickening of the individual branches was observed (Figure 4b–d), implying that MS substitution or interfacial interaction occurred preferentially on the BVO surface. The FESEM images distinctly reveal that MS is dispersed as sheet-like nanostructures anchored along the BVO dendrites, forming a uniform and intimate interfacial junction between the two phases. This strong interfacial coupling between BVO and MS is anticipated to induce synergistic effects that enhance charge carrier dynamics. Specifically, it can promote directional charge transfer across the heterojunction interface, suppress electron-hole recombination, and simultaneously expose a greater number of catalytically active sites. Consequently, such an optimized microstructural configuration is expected to significantly improve the overall catalytic efficiency, redox kinetics, and electrochemical stability of the BVO-MS heterostructures under operational conditions.

**Figure 5:**
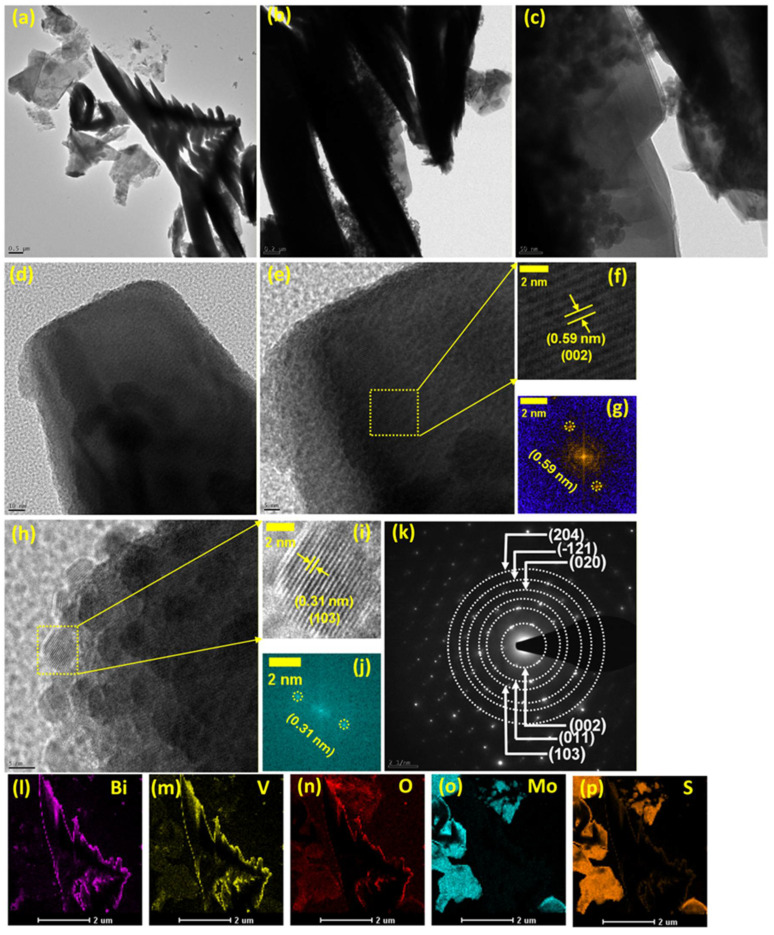
TEM and HR-TEM analysis of BVO-MS10 composite, (**a**–**c**) TEM images at different magnification, (**d**,**e**) HR-TEM images of BVO dendrite, (**f**,**g**) interplanar spacing for (002) plane and FFT pattern, respectively, (**h**) HR-TEM image, (**i**,**j**) interplanar spacing for (103) plane and FFT pattern, respectively, (**k**) SAED pattern, (**l**–**p**) elemental mapping of Bi, V, O, Mo, and S, respectively.

Figure 5 illustrates the TEM and HRTEM images of the BVO-MS10 heterostructure, providing detailed insight into its nanoscale architecture and phase interconnectivity. As depicted in Figure 5a–c, the composite exhibits a well-coupled and coherent morphology, wherein ultrathin sheet-like MS domains are uniformly distributed and conformally anchored onto (opposite side-see Figure 5a–c) the dendritic skeleton of BiVO_4_ (BVO). This structural configuration implies the successful formation of a continuous and intimate heterointerface between the two components, which is crucial for optimizing charge transfer and enhancing the interfacial electrochemical interactions within the system. HRTEM images of the BVO dendritic region are depicted in Figure 5d,e. Upon investigating Figure 5e using Image J software (version 1.51 J), it was revealed that the lattice fringes existing here have an interplanar distance of 0.59 nm, which corresponds to the d-spacing value of the (002) plane. The zoomed view of this lattice plane was represented in Figure 5f, while the Fast Fourier Transform (FFT) pattern was shown in Figure 5g. Similarly, focused on the in-depth part of the BiVO_4_ dendrite as shown in Figure 5h, it was noted that the dendrite formation is a result of the small crystallites/particles. Detailed examination of this image reveals a distinct and well-resolved lattice fringe with an interplanar distance of approximately 0.31 nm, which corresponds to the (103) crystallographic plane of monoclinic BiVO_4_. This observation provides direct evidence of the high crystallinity and structural order within the BVO domains. The corresponding FFT pattern (Figure 5i) further corroborates this finding by displaying sharp diffraction spots consistent with the (103) lattice orientation, as highlighted in Figure 5j. Together, these results confirm the preservation of the crystalline phase and validate the structural integrity of BiVO_4_ within the BVO-MS heterostructure. This observation confirms the high degree of crystallinity retained in the BVO domains after heterostructure formation. Conversely, the HRTEM image focuses only on the MoS_2_ sheet area, which is attached to the opposite side of the dendrites, were represented in Figure S5. Figure S5a illustrates a MoS_2_ sheet well attached to the BiVO_4_ dendrite. Investigating the HR-TEM image in Figure S5b, it was found that it exhibits the lattice fringe pattern with a distance of 0.61 nm, which corresponds to the prominent peak of the few-layered MoS_2,_ i.e., (002) crystal plane. The zoomed area of this fringe pattern was illustrated in Figure S5c with the FFT pattern in Figure S5d. Another highly intense crystal plane of the MoS_2_ was also noted through investigating the HR-TEM of these sheets, which is (103). HR-TEM image in Figure S5e reveals the existence of this plane with the zoomed region in Figure S5f and FFT pattern in Figure S5g. This reveals the presence of crystalline few-layered MoS_2_, as the similarity in the crystal planes was noted in the XRD patterns of pure few-layered MoS_2_, as represented in Figure S1.

The selected area electron diffraction (SAED) pattern (Figure 5k) displays sharp and concentric diffraction rings, reflecting the polycrystalline character of the BVO framework. Such a configuration suggests multiple crystallite orientations, which can contribute to isotropic charge transport and enhanced electrochemical reactivity. Elemental mapping analyses, Figure 5l–p, further corroborate the homogeneous spatial distribution of Bi, V, O, Mo, and S throughout the composite matrix. This uniform dispersion of elements provides strong evidence for the effective integration of the BVO and MS phases, confirming the successful synthesis of a compositionally consistent heterostructure without observable phase segregation or elemental clustering.

**Figure 6:**
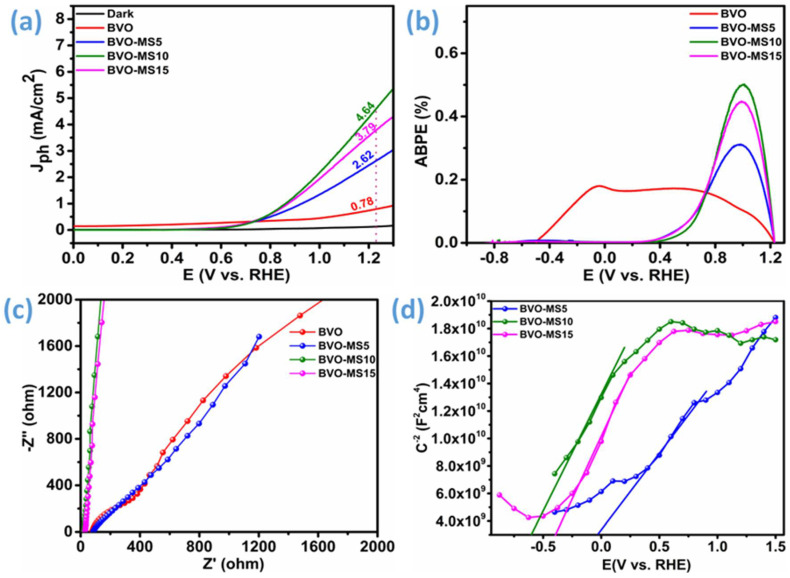
Photoelectrochemical analysis, (**a**) I-V curve of BVO and BVO-MS composites, (**b**) ABPE of BVO and BVO-MS composites, (**c**) EIS spectra of BVO and BVO-MS composites, and (**d**) Mott-Schottky plots of BVO-MS composites.

The PEC performance of BVO electrodes modified with different MS loadings (5%, 10%, and 15%) was tested in 0.5 M Na_2_SO_4_ solution under simulated AM 1.5G light illumination (Figure 6). The assessment of photocurrent density for sulfite oxidation is essential to measure, as it provides insight into the PEC performance of BVO-based electrodes, irrespective of their sluggish water oxidation kinetics. Sulfite oxidation is thermodynamically more favorable than water oxidation. During sulfite oxidation, charge transfer to the electrolyte interface occurs rapidly, making surface charge recombination almost negligible. As shown in Figure 6a, the pure BVO electrode shows the photocurrent densities of 0.78 mA/cm^2^ at 1.23 V vs. RHE. Decoration of MS has been found to significantly elevate the photocurrent densities to 2.62 mA/cm^2^, 4.64 mA/cm^2^, and 3.79 mA/cm^2,^ for BVO-MS5, BVO-MS10, and BVO-MS15, which are much higher than the previous reports, indicating good crystallinity and electron transfer across the interface [19,35,40,41,42,43,44,45]. Furthermore, Table S2 provides an organized comparison of the photocurrent density achieved in this work with values previously reported in the literature. Among them, the photocurrent density of BVO-MS10 is highest, indicating the optimal deposition levels of MS, indicating an optimal test electrode, and further increasing the MS contents resulted in lowering the photocurrent density. This might be due to the shielding effect, blocking the light absorption properties of base materials while hampering the BVO-MS heterojunction interface effect.

Applied bias photo-to-current efficiency (ABPE) of the BVO-based electrodes was calculated from LSV curves (Figure 6b) and according to Equation (1).

(3) $$ABPE\;\left(\%\right)=\frac{J_{ph}\;\left(\frac{mA}{cm^{2}}\right)\times \left(1.23-\left|V_{app}\right|\right)\;\left(V\right)}{P_{light}\;\left(\frac{mW}{cm^{2}}\right)}\times 100\%$$

where *J_ph_* is the photocurrent density, $\left|V_{app}\right|$ is the applied bias, *P_light_* is the power density of the illuminating light source. The BVO-MS10 electrode shows a relatively larger ABPE of 0.49% at a relatively lower applied potential of ~1 V vs. RHE, with the presence of the hole scavenger demonstrating beneficial characteristics of such electrodes for PEC devices. These findings suggest that the synergistic contribution of MS and BVO can help improve the photoelectrochemical water splitting, as evidenced by improved ABPE. The charge transfer kinetics of the synthesized BVO-based electrodes are investigated using EIS under light illumination (AM 1.5G) (Figure 6c). The smaller semicircle radius in Nyquist’s plots is generally related to the charge transfer resistance (*R*_CT_), and a smaller semicircle radius corresponds to a lower charge transfer resistance. As seen from Figure 6c, with the inclusion of MS, the semicircle radius is reduced significantly, demonstrating better electron transfer characteristics. These results further support the earlier result that increasing photocurrent density is due to increased electron transfer across the BVO-MS interface and heterojunction formation, which can assist in effective charge separation.

Mott-Schottky (M-S) measurements were systematically carried out to evaluate the flat-band potentials of the BVO-MS heterostructured electrodes, as presented in Figure 6d. The M-S plots for BVO-MS5, BVO-MS10, and BVO-MS15 exhibit a clear positive slope, confirming their intrinsic n-type semiconducting nature. In n-type semiconductors, the energy separation between the conduction band minimum and the flat-band potential is typically minimal. A photoelectrode exhibiting minimal surface charge recombination typically shows a more negative photocurrent onset potential, reflecting enhanced interfacial charge-transfer kinetics [46,47]. Notably, the extrapolated x-intercepts of the M-S plots indicate a shift in the flat-band potential toward more negative values with increasing MS incorporation up to the optimal composition (BVO-MS10), followed by a slight positive shift for BVO-MS15. The estimated values were approximately +0.07 V, −0.58 V, and −0.38 V (vs. RHE) for BVO-MS5, BVO-MS10, and BVO-MS15, respectively. The pronounced negative shift observed for BVO-MS10 signifies an upward movement of its conduction band minimum, thereby endowing photogenerated electrons with enhanced reducing power and facilitating their migration across the heterointerface. This favorable band alignment establishes a strong internal electric field at the BVO/MS junction, effectively promoting charge separation and directional carrier transport [48]. Consequently, the optimized BVO-MS10 heterostructure achieves superior photoelectrochemical performance owing to its efficient charge separation and accelerated interfacial electron transfer.

**Figure 7:**
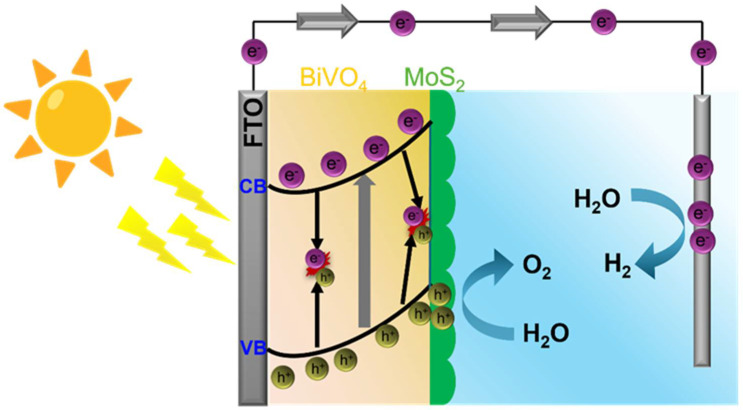
Schematic representing the photoexcitation process of BiVO_4_/MoS_2_ photoanode.

The remarkable enhancement in photoelectrochemical activity and oxygen evolution performance of the BiVO_4_-MoS_2_ photoanode, particularly at the optimized 10 wt% MoS_2_ loading, arises from the efficient interfacial charge transfer and separation governed by the built-in electric field across the heterojunction interface. As shown in Figure 7, the PEC mechanism of the BiVO_4_/MoS_2_ heterojunction photoanode is governed by the synergistic interactions at the BiVO_4_–MoS_2_ interface, which effectively mitigate the intrinsic limitations of pristine BiVO_4_, including rapid electron–hole recombination and sluggish oxygen evolution reaction (OER) kinetics. Under visible-light illumination, BiVO_4_ acts as the primary light-absorbing semiconductor due to its suitable bandgap (~2.4 eV), where incident photons excite electrons from the valence band (VB) to the conduction band (CB), generating electron–hole pairs. The photogenerated electrons in the CB of BiVO_4_ are driven by the built-in electric field and the downward band bending at the FTO/BiVO_4_ interface, facilitating their migration toward the conductive FTO substrate. These electrons are then collected by the external circuit and transported to the counter electrode, where they reduce protons to generate molecular hydrogen. Simultaneously, the photogenerated holes in the VB of BiVO_4_ are efficiently funneled toward the BiVO_4_/MoS_2_ interface due to the favorable band alignment, where MoS_2_ functions as a highly conductive co-catalyst with abundant active edge sites. MoS_2_ serves a dual role: it provides a low-resistance pathway for hole extraction from BiVO_4_, thereby minimizing interfacial recombination, and it catalyzes the water oxidation reaction by lowering the overpotential required for OER. The strong electronic coupling at the heterointerface promotes ultrafast charge separation, as evidenced by the significant reduction in photoluminescence (PL) intensity of the composite, which indicates suppressed electron–hole recombination relative to pristine BiVO_4_. Furthermore, Mott–Schottky analysis reveals that the incorporation of MoS_2_ induces a negative shift in the flat-band potential (Efb) of BiVO_4_, which strengthens the thermodynamic driving force for electron injection toward the FTO and enhances interfacial charge transfer. Electrochemical Impedance Spectroscopy (EIS) further confirms that the heterostructure exhibits a markedly reduced charge-transfer resistance (RCT), highlighting the accelerated interfacial kinetics enabled by MoS_2_. Additionally, the presence of MoS_2_ can extend the light-harvesting range slightly and provide additional active sites for OER, further improving the surface reaction kinetics. Overall, the BiVO_4_/MoS_2_ heterojunction demonstrates a cooperative effect: BiVO_4_ ensures efficient visible-light absorption, MoS_2_ facilitates rapid hole extraction and catalysis at the electrolyte interface, and the heterointerface suppresses electron–hole recombination. These factors collectively lead to enhanced photocurrent density, improved applied bias photon-to-current efficiency (ABPE), and superior PEC water oxidation performance for the optimized BiVO_4_/MoS_2_ (10 wt%) photoanode. To determine whether the sample underwent any structural modification, the XRD patterns and FE-SEM images of the BVO-MS10 film were compared before and after the PEC measurements, as illustrated in Figures S6 and S7, respectively. Both XRD and FE-SEM reflect that there is no remarkable change in the phase or structure of the BiVO_4_/MoS_2_ composite.

## 4. Conclusions

In summary, the in situ engineered BiVO_4_–MoS_2_ (BVO–MS) heterostructure demonstrates a significant enhancement in photoelectrochemical water-splitting performance compared to pristine BVO. The strong interfacial coupling and uniform integration of MS nanosheets effectively facilitate charge transfer, suppress recombination, and improve light absorption. Mott–Schottky analysis confirms that the pronounced negative shift in flat-band potential for the optimized BVO–MS10 electrode results in a favorable band alignment and the formation of an internal electric field that promotes efficient charge separation and transport. Consequently, BVO–MS10 achieves a high photocurrent density and improved ABPE, underscoring its potential as a highly efficient and stable photoanode for sustainable solar-driven hydrogen production.

## Data Availability

The original contributions presented in this study are included in the article/Supplementary Materials. Further inquiries can be directed to the corresponding authors.

## Supplementary Materials

The following supporting information can be downloaded at: https://www.mdpi.com/article/10.3390/ma18245639/s1, Figure S1: X-ray diffraction patterns of bulk MoS2 powder, few-layer MoS2 collected at intermediate, and few-layer MoS2 collected finally; Figure S2: Williamson-Hall (βcosθ vs. 4sinθ) plots of BVO-MS composites at various MS component concentration; Figure S3: Survey spectra of BVO-MS10 composite; Figure S4: FE-SEM images of BVO and BVO-MS composites at lower magnification; Figure S5: HR-TEM analysis of MoS2 sheets connected with BiVO4 dendrites; Figure S6: X-ray diffraction patterns of the FTO, BVO-MS10 sample on FTO before and after PEC measurements; Figure S7: FE-SEM images of the BVO-MS10 sample on FTO. Table S1: Parameters eastimated from X-ray diffraction analysis of BVO-MS samples; Table S2: Comparative analysis of synthesis and PEC performances of several BiVO4-based heterostructure photoanodes.
