# Supplementary material for: Enhanced Photoelectrochemical Performance of BiVO4 Photoanodes Through Few-Layer MoS2 Composite Formation for Efficient Water Oxidation
Source: Materials (Basel). 2025 Dec 15;18(24):5639. doi: 10.3390/ma18245639 (PMC12734439; doi:10.3390/ma18245639)
Supplement: Supplementary file 1 [file materials-18-05639-s001.zip › materials-4000797-supplementary.pdf]

Supplementary files

Enhanced Photoelectrochemical Performance of BiVO4 Photoanodes through Few-Layer MoS_2 Composite Formation for Efficient Water Oxidation

Deepak Rajaram Patil[1,2], Santosh S. Patil[3], Rajneesh Kumar Mishra[*4], Sagar M. Mane[*5], Seung Yoon Ryu[*1,2]

[1]Department of Physics, Dongguk University, Seoul 04620, Republic of Korea.
[2]Photoenegy Harvesting and Conversion Technology (phct), Dongguk University, Seoul 04620, Republic of Korea.
[3]Dr. Vishwanath Karad MIT World Peace University (MIT-WPU), Kothrud, Pune, 411038, India.
[4]Department of Physics, Yeungnam University, Gyeongsan 38541, Gyeongbuk, Republic of Korea
[5]Department of Fiber System Engineering, Yeungnam University, Gyeongsan 38541, Gyeongbuk, Republic of Korea.
* Correspondence: author email: rajneeshmishra08@gmail.com (Rajneesh Kumar Mishra); manesagar99@gmail.com (Sagar M. Mane); justie74@dongguk.edu (Seung Y. Ryu)

Preparation of Few-Layered MoS_2

Bulk MoS_2 powder was exfoliated to obtain few-layer MoS_2 nanosheets using a liquid-phase exfoliation method. The process consisted of two main steps: prolonged sonication followed by sequential centrifugation at low and high speeds. Based on a previously reported procedure [21-main text], 400 mg of bulk MoS_2 powder was dispersed in 200 mL of N, N-dimethylformamide (DMF). The dispersion was sonicated for 20 hours in a bath sonicator (Mujigae, LK-U065D, 40 kHz), with the temperature maintained below 50 °C using an ice bath. After sonication, the resulting gray suspension was first centrifuged at 2500 rpm for 20 minutes to remove larger few-layer particles. The remaining brownish-gray supernatant was then centrifuged at 12,000 rpm for 20 minutes to collect the thinner few-layer MoS_2 nanosheets. The MoS_2 samples obtained from both centrifugation steps were characterized by X-ray diffraction (XRD), as shown in Figure S1, to confirm successful exfoliation. Compared with bulk MoS_2, the sample from the first centrifugation displayed reduced peak intensities and the disappearance of several diffraction peaks, indicating partial exfoliation. After the second, higher-speed centrifugation, most diffraction peaks further diminished, with only a few strong reflections, primarily the (002) plane and minor

contributions from the (100), (103), and (105) planes, remaining. This confirms the formation of a few-layer MoS_2 nanosheet. The resulting few-layer MoS_2 nanosheets were subsequently used to develop composite photoanodes with $BiVO_4$.

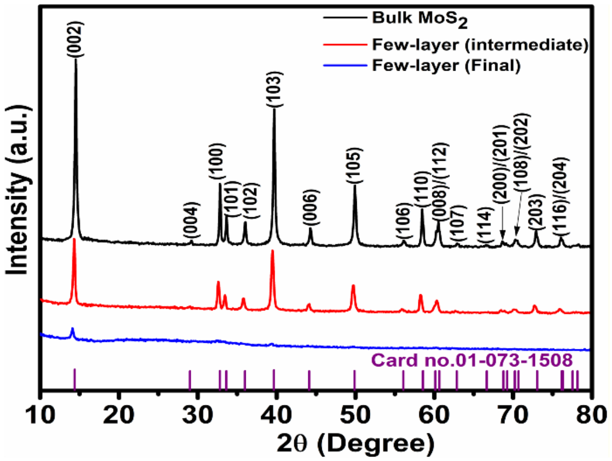

Figure S1. X-ray diffraction patterns of bulk MoS_2 powder, few-layer MoS_2 collected at intermediate, and few-layer MoS_2 collected finally.

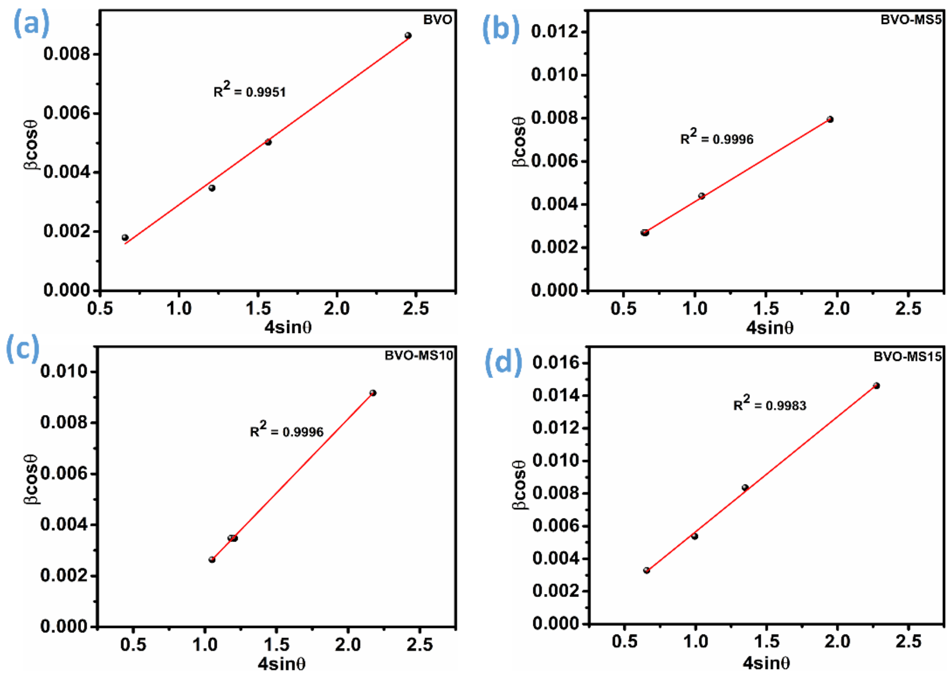

Figure S2. Williamson-Hall (βcosθ vs 4sinθ) plots of BVO-MS composites at various MS component concentration.

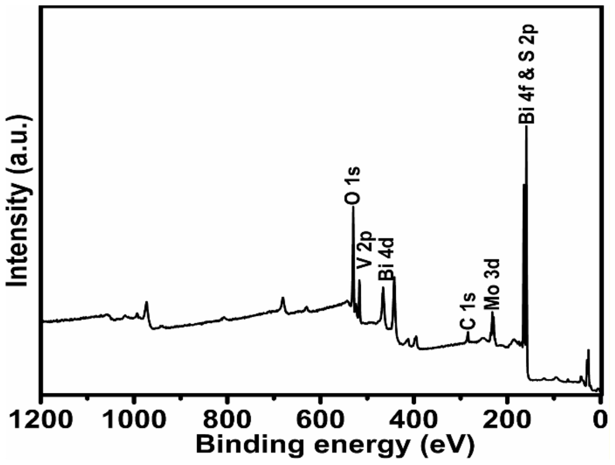

Figure S3. Survey spectra of BVO-MS10 composite.

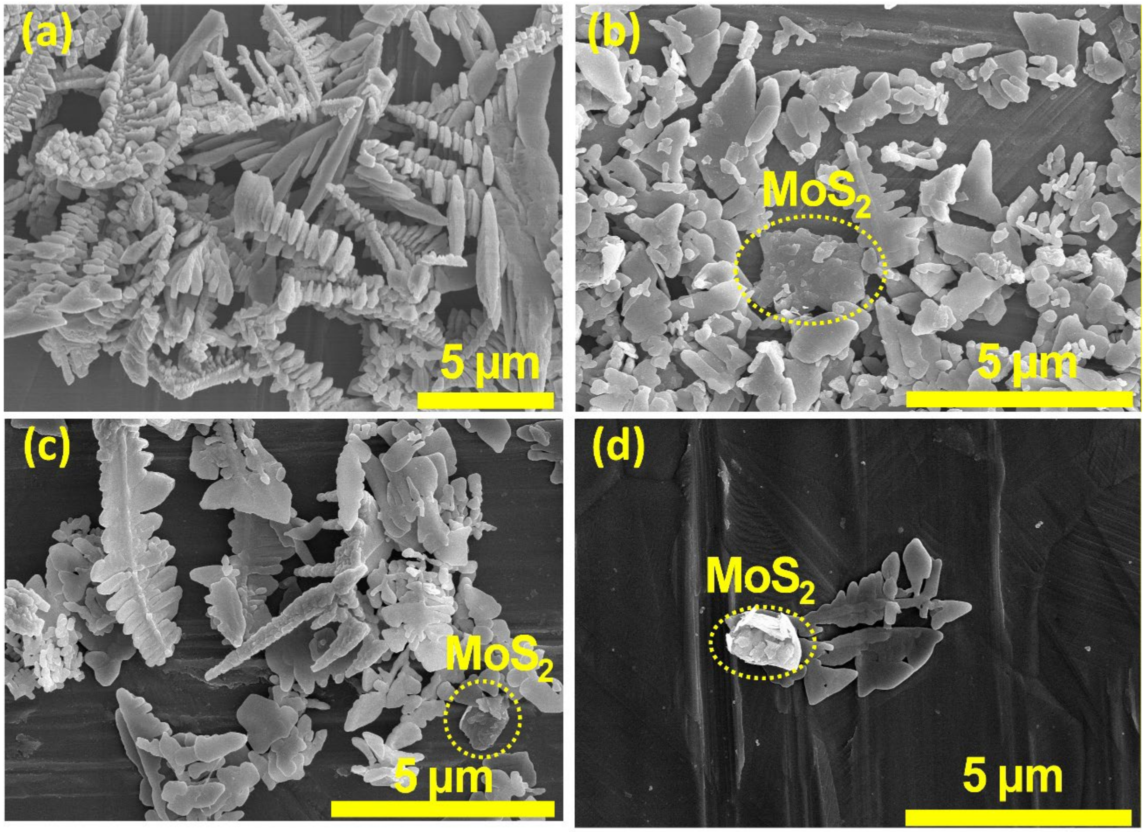

Figure S4. FE-SEM images of BVO and BVO-MS composites at lower magnification, (a) BVO, (b) BVO-MS5, (c) BVO-MS10, and (d) BVO-MS15.

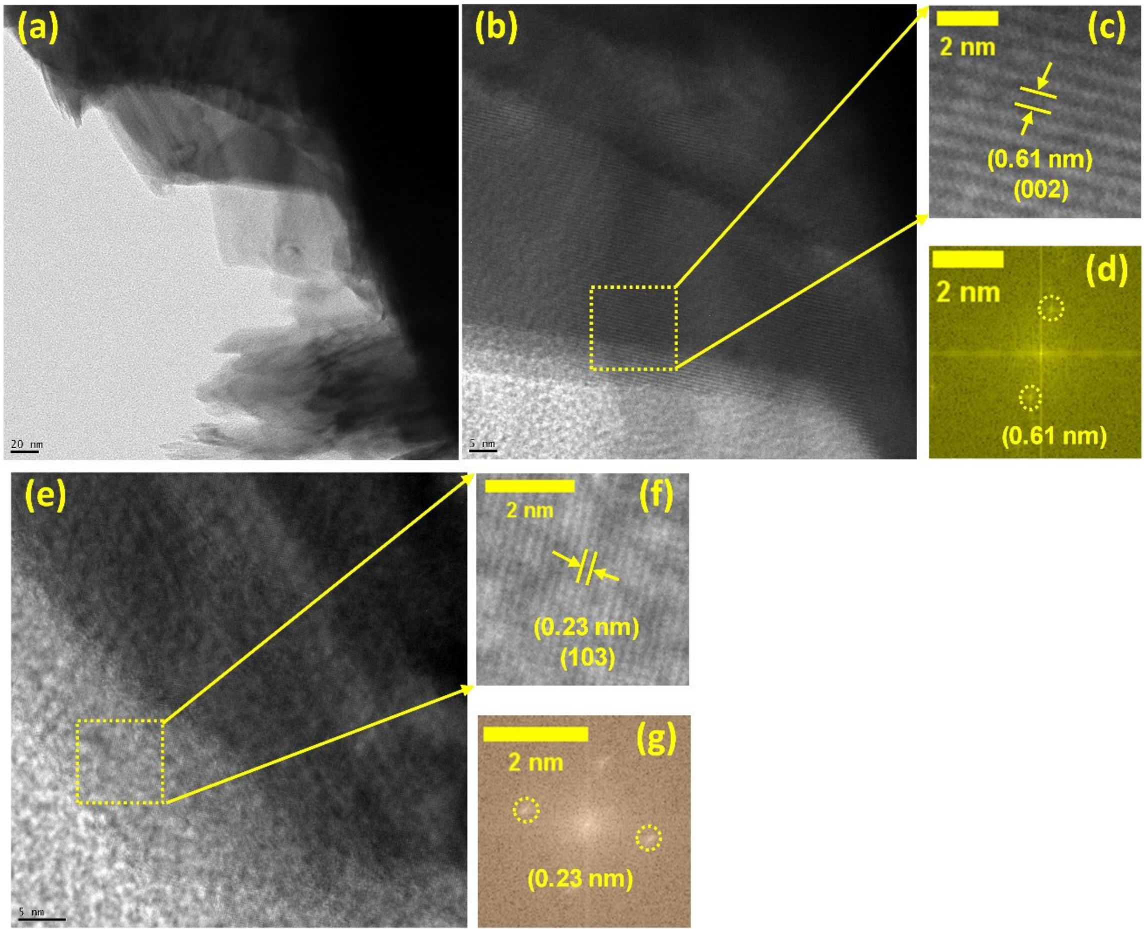

Figure S5. HR-TEM analysis of MoS2 sheets connected with BiVO4 dendrites, (a) HR-TEM reflecting part of dendrite connected with MoS2 nanosheet, (b) HR-TEM image of sheet reflecting (002) plane, (c & d) interplanar spacing of (002) plane and FFT pattern, (e) HR-TEM image of sheet reflecting (103) plane, and (f & g) interplanar spacing of (103) plane and FFT pattern.

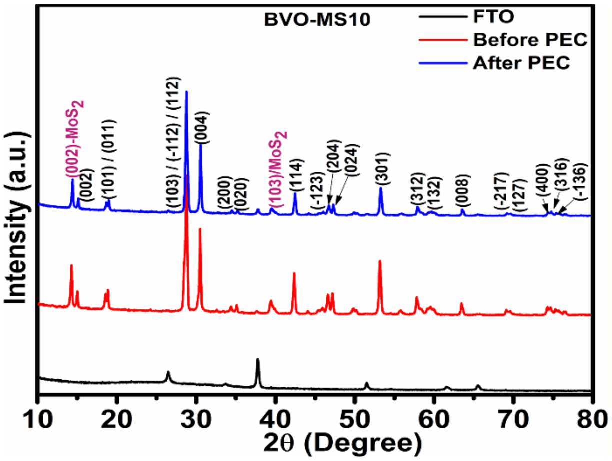

Figure S6. X-ray diffraction patterns of the FTO, BVO-MS10 sample on FTO before and after PEC measurements.

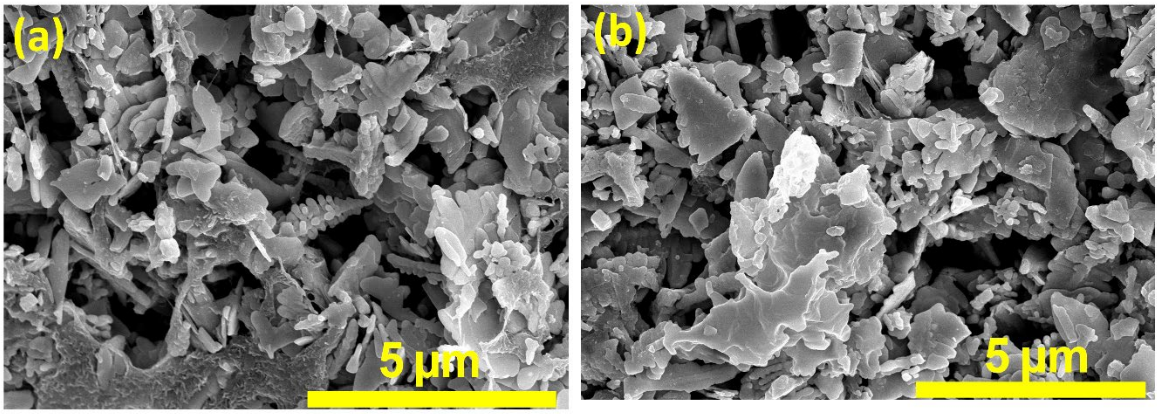

Figure S7. FE-SEM images of the BVO-MS10 sample on FTO, (a) before PEC measurements, and (b) after PEC measurements.

Table S1. Parameters eastimated from x-ray diffraction analysis of BVO-MS samples.

Sample name	Lattice parameters (Å)			Williamson-Hall method	
	a	b	c	Average crystallite size (D) nm	microstrain (ε)
BVO	5.1928	11.6894	5.0917	43.71	0.00387
BVO-MS5	5.1956	11.7309	5.0911	39.00	0.00403
BVO-MS10	5.1973	11.7314	5.0907	41.53	0.00582
BVO-MS15	5.1964	11.7297	5.0921	50.72	0.00706

Table S2: Comparative analysis of synthesis and PEC performances of several BiVO4-based heterostructure photoanodes.

Heterostructure	Fabrication method	Morphology	Photocurrent @ 1.23 V	ABPE %	ref
MOF/$BiVO_4$	Spin coating/dip coating	nanoparticles	2.43	0.55 @ 0.78	1
N:NiFeOx/$BiVO_4$	electrodeposition/dip coating	worm-like porous structure	6.4	1.9% @ 0.73	2
NiOOH/$BiVO_4$	Photoelectrodeposition	nanoparticles	2.4	-	3
SPNTs/ $BiVO_4$	microwave	nanotubes	0.68	0.05 @ 1.1	4
Co−La Hydroxide /$BiVO_4$	Pulse plating	Nano-micro partic-les	2.02	0.38 @ 0.6	5
Co-Pi/$BiVO_4$	Microwave/drop cast	microcubes	3.5	1.5 @ 0.7	6
rGO/ $BiVO_4$	hydrothermal	dendrite	1	-	
MoS_2/$BiVO_4$	Solution growth/dip coating	Nano-Pyramid	2.0	0.52 @ 0.8	19
MoS_2/$BiVO_4$	hydrothermal	nanosheets	4.02	1.3 @ 0.7	34
MoS_2/$BiVO_4$	In-situ hydrothermal-mal	Dendrite/nanoshe-ets	4.64	0.49 @ 1	present
